# Peripheral T-Cell Lymphomas: A Review of Current Approaches and Hopes for the Future

**DOI:** 10.3389/fonc.2013.00138

**Published:** 2013-05-28

**Authors:** Alan P. Skarbnik, Meher Burki, Barbara Pro

**Affiliations:** ^1^Medical Oncology, Fox Chase Cancer Center, Philadelphia, PA, USA; ^2^Medical Oncology, Kimmel Cancer Center, Thomas Jefferson University Hospital, Philadelphia, PA, USA

**Keywords:** PTCL, ALCL, brentuximab, pralatrexate, romidepsin, alisertib, zanolimumab, bortezomib

## Abstract

Peripheral T-cell lymphomas (PTCL) are a diverse group of lymphoproliferative disorders, which share a common denominator of overall poor prognosis, with few exceptions. In this article, the authors review current standard of care approaches for the treatment of PTCLs, the role of stem-cell/bone marrow transplantation, and current developments in novel targeted therapies.

## Definition and Introduction

Peripheral T-Cell lymphomas (PTCL) are a diverse group of lymphoproliferative disorders with different biological and clinical behavior. Comparatively to their B-cell counterparts, PTCLs are significantly rarer and more difficult to treat, either due to the paucity of large trials carrying the evidence to suggest specific therapeutic approaches, or due to the biology of the disease. When compared to B-cell NHL, the prognosis for PTCL remains poor, mainly due to lower response rates and shorter duration of response to standard combination chemotherapy regimens.

Peripheral T-Cell lymphomas account for 10–15% of all NHLs in North America, and are characterized by the presence of malignant mature T-cells (derived from post-thymic T-cells) or NK cells (Savage, 2007). Some forms of PTCL are more common, or have distinct molecular and clinical features that may dictate therapeutic choices, but, overall, PTCL has classically been treated with regimens adapted from B-cell lymphomas, with generally poor outcomes.

In 2008, the fourth edition of the *World Health Organization (WHO) Classification of Tumors of Hematopoietic and Lymphoid Tissues* (Swerdlow, 2008) was published, and subdivided PTCL even further. While this was helpful in providing clinicians with more accurate information to predict prognosis, it has not translated, so far, into a guide for better therapeutic choices. The *International T-cell and natural killer/T-cell lymphoma study* (Vose et al., 2008) provided insight into the epidemiology of this group of diseases. Based on their results, the most common subtype of mature T-cell lymphomas is PTCL, non-otherwise specified (PTCL, NOS; 26%), followed by angioimmunoblastic T-cell lymphoma (AITL; 18.5%), anaplastic large cell lymphoma, ALK-positive (ALCL, ALK+; 7%), and anaplastic large cell lymphoma, ALK-negative (ALCL, ALK−; 6%). Interestingly, PTCLs show significant variation in geographic and racial distribution. In Asian populations, for instance, PTCLs are responsible for a larger proportion of NHL, which could be a result both from a true increased incidence, as well as a relative decrease of some subtypes of B-cell lymphomas which are more commonly seen in North American and European populations (i.e., follicular lymphoma). Another explanation would be the increased incidence of adult T-cell leukemia/lymphoma (ATL) in regions where the human T-cell lymphotropic virus 1 (HTLV-1) is endemic, such as Japan and the Caribbean.

Peripheral T-Cell lymphomas-NOS is the most common subtype of T-cell lymphoma, usually involving nodal sites, with many patients presenting with extra-nodal involvement of the gastrointestinal system, liver, bone marrow, and spleen. When compared to B-cell NHLs, PTCL-NOS present a poorer overall survival (OS) and progression-free survival (PFS) (Savage et al., 2004). AITL usually presents in older patients, with diffuse lymphadenopathy, many times associated to hepatosplenomegaly, eosinophilia, skin rash, fever, and hypergammaglobulinemia. Prognosis is similar to PTCL-NOS, with 5-year OS rate of 33% (Mourad et al., 2008). ALCL, ALK+ is a CD30-expressing type of PTCL, characterized by the over expression of ALK-1 protein, which results from the chromosomal translocation t (2;5) (Mourad et al., 2008; Swerdlow, 2008). It usually presents in children on younger patients, and carries an overall good prognosis, when compared to its ALK-negative counterpart, which usually presents in older patients. Five-year OS rates following anthracycline-based chemotherapy have been reported as 79% for ALCL, ALK+ and 46% for ALCL, ALK− (Gascoyne et al., 1999).

In this review, we will discuss standard therapeutic approaches for PTCLs, as well as novel therapeutic agents.

## Frontline Therapy

There is no consensus among specialists regarding standard frontline therapy for PTCL.

Commonly, CHOP (cyclophosphamide, doxorubicin, vincristine, prednisone) is the “go-to” chemotherapy regimen of choice for PTCL (Broussais-Guillaumot et al., 2013), despite evidence that, except for ALCL, ALK+, it is largely ineffective, as evidenced by a large retrospective analysis from the German High-Grade Non-Hodgkin Lymphoma Study Group (Schmitz et al., 2010). In this analysis, 343 patients with a diagnosis of PTCL were treated with six to eight cycles of CHOP or CHOP plus etoposide (CHOEP). Three-year event-free survival (EFS) and OS for patients in the four major subtypes were 75.8 and 89.8% for ALK-positive ALCL; 50.0 and 67.5% for AITL; 45.7 and 62.1% for ALK-negative ALCL; 41.1 and 53.9% for PTCL-NOS. In younger patients with normal lactate dehydrogenase (LDH), the addition of etoposide to CHOP significantly improved 3-year EFS (75.4 versus 51.0%, *P* < 0.003), but there was no statistically significant difference in OS. For patients older than 60 years of age, the addition of etoposide did not yield any advantage, mainly due to added toxicities. This analysis also took into consideration patients in trials of dose intensification or dose escalation of CHOP/CHOEP, and found no improvement in OS when compared to standard CHOP given every 21 days.

More intensive chemotherapy regimens, such as hyperfractionated cyclophosphamide, vincristine, doxorubicin, and prednisone (hyper-CVAD) were compared to CHOP in a retrospective analysis by the MD Anderson group (Escalon et al., 2005), and showed no additional benefit. Clinical outcomes for 135 patients with previously untreated PTCL, who received frontline therapy, were evaluated. The estimated 3-year OS for patients receiving CHOP was 62%, while for patients receiving intensive therapy it was 56%. When patients with a diagnosis of ALCL were excluded from the analysis, the 3-year OS was 43% for patients treated with CHOP and 49% for patients treated with intensive therapy. Additionally, the investigators identified parameters that may be independent prognostic factors in PTCL (excluding ALCL), such as ECOG performance status >2, beta-2-microglobulin level >2 mg/L, LDH level higher than normal, bulky disease ≥7 cm, and a higher international prognostic index (IPI).

Bortezomib, a selective inhibitor of the proteasome 26S, involved in the NF-κB cell signaling pathway, has been evaluated for treatment of patients with PTCL. A Phase I study of bortezomib used in association with CHOP chemotherapy was conducted in 13 patients with advanced PTCL or NK/T-cell lymphoma (Lee et al., 2008). The reported CR rate was 62%, with no data published on PFS or OS. A phase II study of bortezomib as a single agent for patients with relapsed PTCL (*n* = 2) or cutaneous T-cell lymphoma (CTCL) (*n* = 13) reported objective response rates (ORR) of 67%, with a duration of response ranging from 7 to 14 months and both patients with PTCL achieving CR (Zinzani et al., 2007).

Recently, results of a Phase II trial combining bortezomib to CHOP chemotherapy in the frontline setting for patients with stage III/IV PTCL were published (Kim et al., 2012). Of the 46 patients enrolled, 30 achieved a CR (65%), with an ORR of 76%. In spite of good initial response rates, the 3-year OS and PFS rates were 47 and 35% respectively, not much different than historical controls using CHOP alone as frontline therapy. Bortezomib remains an attractive agent for prospective clinical trials in patients with PTCL. Given the low rates of significant complications, association of bortezomib with standard chemotherapy should be further studied.

Gallamini et al. (2007) have reported a prospective Phase II trial of frontline CHOP combined to the anti-CD52 monoclonal antibody alemtuzumab (CHOP-C), for 24 patients with a diagnosis of PTCL. Complete remission was achieved in 71% of patients and after a median follow-up of 16 months, median duration of response was 11 months. Two Phase III trials in Europe are currently recruiting patients with PTCL for a prospective comparison between CHOP and CHOP-C as frontline therapy (ACT-1 and ACT-2 trials). Other efforts are underway, attempting to improve response and survival rates combining other novel agents to CHOP.

## The Role of Autologous Stem-Cell Transplant

Given the overall poor outcomes with conventional therapy as frontline treatment for PTCL, the role of high-dose chemotherapy with autologous stem-cell rescue (HDT/ASCR) has been studied as a consolidation option after successful frontline therapy. Several retrospective studies have reported favorable outcomes for consolidation HDT/ASCR, with 3-year OS and PFS ranging from 53 to 58% and 44 to 50%, respectively (Schetelig et al., 2003; Jantunen et al., 2004; Feyler et al., 2007; Kim et al., 2007a; Rodriguez et al., 2007a; Kyriakou et al., 2008; Yang et al., 2009). Prospective studies solidified the role of HDT/ASCR in PTCL by yielding positive outcomes in this patient population. Table 1 depicts some of these studies.

**Table 1:** **Clinical trials of HDT/ASCR for PTCL**.

Reference	Regimen	Number of patients	OS rate	PFS rate
GEL-TAMO (Rodriguez et al., 2007b)	MegaCHOP ×3, followed by HDT/ASCR (in unresponsive patients, addition of ifosfamide + etoposide ×2 prior to HDT/ASCR)	26 (ALCL, ALK + excluded)	73% at 3 years	53% at 3 years
NLG-T-01 (d’Amore et al., 2012)	CHOEP-14 ×6, followed by HDT/ASCR (in patients >60 years of age, etoposide was excluded)	166 (ALCL, ALK + excluded)	51% at 5 years	44% at 5 years
Corradini et al. (2006)	APO ×2 + DHAP ×2, followed by HDT/ASCR or MACOP-B 8 weeks followed by HDT/ASCR	62	34% at 12 years	30% at 12 years
GELCAB (Mercadal et al., 2008)	High-dose CHOP/ESHAP ×6 followed by HDT/ASCR	41 (ALCL, ALK + excluded)	39% at 4 years	30% at 4 years
Reimer et al. (2009)	CHOP ×4–6, followed by HDT/ASCR	83	48% at 3 year (for patients in CR)	36% at 3 year

*CHOEP, cyclophosphamide, doxorubicin, vincristine, etoposide, and prednisone; APO, doxorubicin, vincristine, prednisone; DHAP, dexamethasone, cytarabine, cisplatin; MACOP-B, methotrexate, cytarabine, cyclophosphamide, vincristine, prednisone, and bleomycin; ESHAP, etoposide, methylprednisolone, cytarabine, and cisplatin; HDT/ASCR, high-dose chemotherapy/autologous stem-cell rescue; ALCL, anaplastic large cell lymphoma*.

To date, there are no published randomized trials comparing conventional chemotherapy alone to first-line consolidation with HDT/ASCR, so the true impact of this treatment modality on survival rates has not been completely established. In light of the available data, HDT/ASCR remains a reasonable treatment modality for patients with a good performance status who present a significant response to frontline therapy.

## The Role of Allogeneic Hematopoietic Bone Marrow Transplant

Given the significant relapse rates after frontline therapy for PTCL, even after consolidation with HDT/ASCR in first remission, there has been increasing interest in allogeneic hematopoietic bone marrow transplant (allo-BMT) as a treatment modality, due to its potential in eliciting a graft-versus-lymphoma (GVL) effect. Recently, the Johns Hopkins group reported on 44 consecutive patients who underwent related-donor allo-BMTs for PTCL, including 22 haploidentical grafts (Kanakry et al., 2013). Most patients presented with poor-risk disease, with 60% having received at least two prior chemotherapy regimens, 50% had history of chemorefractory disease and 25% had active disease at the time of allogeneic stem-cell transplantation (allo-SCT). Grafts were marrow derived in 43 of the cases. Patients received either a reduced intensity conditioning (RIC) regimen (*n* = 24) or a myeloablative conditioning (MAC) regimen (*n* = 20). Estimated 2-year PFS for all patients was 40% and OS was 43%. There was a non-statistically significant trend toward better PFS for patients receiving allo-BMT in first remission, versus beyond first remission (53% at 2 years, versus 29%, *p* = 0.08). The 1-year estimated non-relapse mortality (NRM) was 10% for patients receiving MAC and 8% for patients receiving RIC (11% for RIC/haploidentical transplants).

This PTCL-restricted series show that using RIC regimens for patients with high-risk PTCL is feasible, with less NRM when compared to the use of MAC regimens. A remarkable tendency toward better PFS when performing allo-BMT following first remission may help solidify the role of this therapeutic approach for PTCL, although further comparative studies are needed to better elucidate the appropriate timing for allo-BMT. With continuous efforts toward augmentation of GVL effect while minimizing graft-versus-host disease (GVHD) and transplant-related toxicities, especially when using RIC regimens, allo-BMT may play an important role in the management of PTCLs in the near future.

## Treatment for Relapsed/Refractory Disease

Until recently, salvage therapy modalities for relapsed/refractory PTCL were restricted to combination chemotherapy regimens derived from B-cell lymphoma trials, followed by HDT/ASCR or allo-SCT. Second-line chemotherapy with ICE (ifosfamide, cisplatin, etoposide) followed by HDT/ASCR, for example, yielded a median PFS of only 6 months (counted from the last treatment with ICE), with 70% of patients relapsing at 1 year (Horwitz et al., 2005).

For allo-SCT, a retrospective analysis conducted in France revealed 5-year EFS and OS of 53 and 57%, respectively, but with a 5-year transplant-related mortality (TRM) of 34% and an early TRM (<100 days) rate of 21% (Le Gouill et al., 2008). Additional retrospective analyses are available, but none revealed 3-year OS rates higher than 40–45%, with a repeatedly significant early TRM rate across analyses (Smith et al., 2010; Beitinjaneh et al., 2011).

While HDT/ASCR and allo-SCT remain viable treatment options for younger patients whose disease relapsed, a significant response to systemic therapy must be achieved prior to using these consolidation alternatives. Furthermore, appropriate treatment for older or unfit patients is subject of much controversy as high-dose therapy-based consolidation is not an option.

Suboptimal results of B-cell derived therapies led investigators to design clinical trials specifically for PTCL. Given the rarity of the disease and no established standard of care in the relapse setting, most data comes from non-randomized phase II trials or case series. Table 2 depicts current approaches for relapsed/refractory disease.

**Table 2:** **Novel. agents for treatment of PTCL (in the relapsed setting, unless otherwise specified)**.

Agent	ORR (%)	CR (%)	OS/PFS/duration of response (DOR)
**CHEMOTHERAPY**			
Gemcitabine (Zinzani et al., 2010)	60–70	30	DOR 34 months
Clofarabine (Mulford et al., 2010)	21	0	N/A
Forodesine (Duvic et al., 2007)	39	0	DOR 127 days
Nelarabine (Czuczman et al., 2007)	10	0	DOR 1.2 months
Bendamustine (Damaj et al., 2013)	50	28 (after three cycles)	DOR 3.5 months
**IMMUNOTHERAPY**			
Denileukin diftitox (Dang et al., 2007)	48	22	PFS 6 months
Denileukin diftitox + CHOP (Foss et al., 2013) (frontline)	65	N/A	DOR 30 months
			PFS 12 months
**FOLATE ANALOGS**			
Pralatrexate (O’Connor et al., 2011)	29	11	PFS 3.5 months
			OS 14.5 months
**HISTONE DEACETYLASE INHIBITORS**			
Romidepsin (Coiffier et al., 2012)	25	15 CR/CRu	DOR: 89% sustained CR at 13.4 months
Belinostat (Zain et al., 2010)	32	N/A	DOR 8 months
**ANTIBODIES**			
Brentuximab vedotin (for ALCL) (Pro et al., 2012)	86	57	DOR 12.6 months
Zanolimumab (d’Amore et al., 2010)	21	N/A	N/A
Alemtuzumab (low dose) (Zinzani et al., 2005)	50	33	N/A
Mogamulizumab (for ATL) (Ishida et al., 2012)	50	30.7	PFS 5.2 months
			OS 13.7 months
**PROTEASOME INHIBITORS**			
Bortezomib + CHOP (frontline) (Kim et al., 2012)	67–76	62–65	3-year OS 47%
			3-year PFS 35%
**IMMUNOMODULATORY DRUGS**			
Lenalidomide (Dueck et al., 2010)	30	0	PFS 96 days
**AURORA KINASE INHIBITORS**			
Alisertib (Friedberg et al., 2011)	57	N/A	N/A
**OTHER DEPSIPEPTIDES**			
Plitidepsin (Ribrag et al., 2013)	20.7	CR 6.8	N/A
		PR 13.8	
**TKIs**			
Dasatinib (William et al., 2010)	32 (for all NHLs enrolled)	CR: two patients with PTCL	2-year PFS 13% (for all patients)
		PR: one patient with PTCL	2-year OS 50% (for all patients)

### Chemotherapy

In studies with heavily pretreated patients with a diagnosis of PTCL, gemcitabine has shown activity as monotherapy, yielding ORR of 60–70% (Zinzani et al., 1998; Sallah et al., 1999). A recent report on the long-term follow-up of patients with relapsed PTCL treated with gemcitabine as monotherapy confirmed the activity of this agents, with a CR and PR rate in the PTCL-NOS population of 30 and 25%, respectively (Zinzani et al., 2010). Five out of 20 patients with PTCL-NOS presented continuous CR at the time of final follow-up, with a median duration of CR of 34 months (range 15–60 months).

Clofarabine is a purine nucleoside analog that has been reported as being active in the treatment of PTCL. A recent interim report on a phase I/II study of clofarabine as monotherapy for patients with relapsed/refractory PTCL has shown a ORR of 21%, with mild and manageable side effects (Mulford et al., 2010).

Forodesine is a novel purine nucleoside analog, which inhibits the enzyme purine nucleoside phosphorylase, responsible for cleaving deoxyguanine to guanine. With deactivation of this enzyme, deoxyguanine is instead converted to deoxyguanosine triphosphate (dGTP), leading intracellular accumulation of this compound, causing apoptosis and reduced proliferation of lymphocytes. This is a unique mechanism of action among purine nucleoside analogs, which usually exert their antineoplastic function through interaction with nucleic acids (Kicska et al., 2001). Forodesine has been shown to selectively inhibit T-lymphocytes, and was investigated as an agent for CTCL. In a phase I/II study (Duvic et al., 2007), patients received oral forodesine at escalated doses. Although a maximum tolerated dose (MTD) was not reached, the optimal biological dose was determined as being 80 mg/m^2^ daily. Thirty-six patients received the optimal dose, yielding an ORR of 39% with a median duration of response of 127 days. To date, there is no published data on forodesine used for non-cutaneous PTCL.

Nelarabine, a pro-drug of arabinofuranosylguanine (ara-G), is toxic to mature T-cells and immature T-lymphoblasts, and has been approved by the FDA for use in the treatment of relapsed or refractory T-ALL and T-lymphoblastic lymphoma. This agent has been investigated in a phase II trial, in adults with relapsed/refractory PTCL or systemically untreated CTCL (Czuczman et al., 2007). Out of 19 patients, only 2 presented partial remission, and median EFS was 1.2 months. Additionally, 33% of patients experienced Grade 3 or 4 neurologic toxicities, deeming this agent too toxic and with low efficacy in the treatment of CTCL or PTCL as monotherapy.

Recently, an open label, prospective phase II trial reported on the use of bendamustine for patients with relapsed/refractory PTCL (Damaj et al., 2013). Bendamustine is a “dual-structure” drug, with an alkylating portion, derived from nitrogen mustard, and a purine analog portion. Bendamustine does not present cross-resistance with other alkylating agents, and only presents partial cross-resistance with other DNA binding agents. In this trial, patients with relapsed PTCL or CTCL stage IIB or higher (excluding Sezary syndrome) who received three or less lines of systemic therapies, were enrolled, to receive bendamustine 120 mg/m^2^ per day over 30–60 min on days 1 and 2 every 3 weeks, for a total of six cycles. Of the 60 enrolled patients, 15 received the planned six cycles of bendamustine, 25 patients (42%) received three to five cycles, and 20 patients (33%) received fewer than three cycles. Early discontinuation occurred either due to progressive disease (PD) (53%) or significant side effects from therapy. ORR achieved after three cycles of bendamustine was 50%, including CR/CRu of 28% and PR of 22%. Three patients had stable disease, and 27 patients (45%) had PD. Four patients with PR after three cycles converted their response to CR after six cycles. Median duration of response was 3.5 months, with 30% of responses lasting more than 6 months. This study confirms the efficacy of bendamustine as a single agent for relapsed PTCL, and paves the way for future studies incorporating bendamustine into multi-drug regimens.

### Denileukin diftitox

Denileukin diftitox, a genetically engineered drug combining the full human sequence of IL-2 with the cytotoxic and membrane-translocating domains of the diphtheria toxin, was tested in a phase II study for patients with relapsed/refractory PTCL (*n* = 27), yielding ORR of 48%, with a CR rate of 22% and a median PFS of 6 months (Dang et al., 2007). When responses were evaluated based on CD25 status, ORR was 61.5% for patients with CD25 positive tumors and 45.5% for patients with CD25 negative tumors. Adverse events in this study were mild and transient.

The recently published phase II CONCEPT trial enrolled 49 patients with newly diagnosed PTCL. Patients received denileukin diftitox with CHOP every 21 days, for six to eight cycles (Foss et al., 2013). In the intent-to-treat population, ORR was 65%, with a median duration of response of 30 months, and median PFS of 12 months. OS rate at the end of the study was 63.3% (median OS not achieved). There were three treatment related deaths, and most grade 3 or higher adverse events were hematologic.

### Folate analogs

Pralatrexate, a novel folate analog, is designed to be internalized by the reduced folate carrier (RFC), an oncofetal protein that regulates internalization of natural folates required for biosynthesis of DNA bases, and is expressed on both embryonic and malignant tissues. Pralatrexate has a high affinity for RFC, which may lead to selective tumor accumulation (Wang et al., 2003). Early phase I/II trials of pralatrexate in patients with relapsed B- or T-cell lymphomas established its efficacy in this setting, yielding an ORR of 54% for patients with PTCL (O’Connor et al., 2009).

A large, pivotal phase II study (PROPEL study) established the role for pralatrexate in relapsed/refractory PTCL (O’Connor et al., 2011). One hundred and fifteen patients, across 25 different centers, were enrolled in this study, with 111 patients receiving pralatrexate. All patients had received at least one previous line of therapy for PTCL. Treatment regimen consisted of pralatrexate 30 mg/m^2^ weekly for 6 out of 7 weeks, continuing until disease progression or unacceptable toxicities. Overall response rate was 29%, including 11% CRs, with median PFS and OS of 3.5 and 14.5 months respectively. Although supplementation with folic acid and vitamin B12 was provided, a significant rate of grade 3/4 mucositis (22%) and cytopenias occurred (32% thrombocytopenia, 22% neutropenia, and 18% anemia). Pralatrexate was the first FDA-approved single agent for the treatment of patients with relapsed/refractory PTCL.

### Histone deacetylase inhibitors

Increased histone acetylation has been shown to induce antitumor activity through increased tumor suppressor gene transcription, growth inhibition, cell cycle regulation, and apoptosis (Cress and Seto, 2000; Santini et al., 2007). Romidepsin is a potent histone deacetylase (HDAC) inhibitor, functioning at nanomolar concentrations, and shown to have activity in PTCL in phase I and II trials (Piekarz et al., 2001, 2011). A large, international, pivotal phase II study of romidepsin in relapsed/refractory PTCL was conducted, enrolling 131 patients, with a primary endpoint of assessing the rate of complete responses (Coiffier et al., 2012). ORR was reported at 25%, with a 15% rate of CR/CRu (Complete response, unconfirmed) as assessed by an independent review committee. Of the patients who achieved CR/CRu, 89% had a sustained response, without evidence of progression of disease at a median follow-up of 13.4 months. Grade 3/4 toxicities were mainly hematologic and manageable with blood products or growth factor support. This trial led to the FDA-approval of romidepsin as therapy for patients with relapsed/refractory PTCL.

Based on the positive results achieved with romidepsin, additional HDAC inhibitors are being studied in PTCL. Belinostat, a potent class I and II HDAC inhibitor, is being studied in a phase II clinical trial (ClinicalTrials.gov Identifier: NCT00865969) for patients with PTCL whose disease progressed after at least one line of systemic therapy. At a dose of 1000 mg/m^2^ IV for days 1–5 of a 21-day cycle, the interim efficacy report for this trial has revealed an ORR of 32% with a median duration of response of 8 months (Zain et al., 2010). Panobinostat is a pan-HDAC inhibitor, tested across a spectrum of hematological malignancies, with few PTCL patients receiving this drug, although some responses have been reported in this patient population (Ottmann et al., 2008). Vorinostat is a HDAC inhibitor approved for use in CTCL, without any trials published, to date, of its use in PTCL.

### Antibody-directed therapy

The pilot study for alemtuzumab, a monoclonal antibody directed to CD52 (a molecule highly expressed by malignant T-cells), in relapsed/refractory PTCL used a rapidly escalating dosage schedule during the first week of treatment, to achieve a maximum dose of 30 mg intravenously three times per week for a maximum of 12 weeks (Enblad et al., 2004). Of the 14 patients enrolled in the study, 5 (36%) showed some degree of response, with 3 (21%) CRs, although the treatment was deemed too toxic, with cytomegalovirus reactivation in 6 patients, pulmonary aspergillosis in 2 patients, Epstein–Barr virus-related hemophagocytosis in 2 patients, significant pancytopenia in 4 patients, and 5 patients dying of causes related to treatment. A subsequent Phase II study of reduced-dose alemtuzumab (10 mg three times/week, for 4 weeks) in patients with relapsed PTCL (*n* = 10; PTCL-NOS, *n* = 6) showed a ORR of 50% (CR 33%) in the population subset of patients with PTCL-NOS, with significantly less toxicities than the pilot study (Zinzani et al., 2005). This has established alemtuzumab as a viable treatment option for relapsed/refractory PTCL.

Zanolimumab is a fully human monoclonal antibody targeted to the CD4 receptor, a molecule found in most T-lymphocytes. This drug induces antibody-dependent cell cytotoxicity (ADCC), direct apoptosis of CD3/CD4 positive cells and decreases T-cell activation by interfering with CD4-MHC class II interaction. A phase II multicenter study investigated zanolimumab in 21 patients with relapsed/refractory PTCL (d’Amore et al., 2010). Based on previous data of CTCL (Kim et al., 2007b), the investigators used a dose of 980 mg weekly. ORR was reported at 21%, with two unconfirmed CRs. Patients tolerated zanolimumab well, with most serious adverse events being related to infusion reactions.

Siplizumab is a human monoclonal antibody targeted to CD2, a molecule found in human T-lymphocytes and NK cells. A phase I trial of this drug in T-cell lymphoproliferative disorders was initiated, and although initial reports (O’Mahony et al., 2005) found siplizumab to be well tolerated and a MTD was not reached, follow-up reports found an increased rate of Epstein–Barr virus-related lymphoproliferative disorders (O’Mahony et al., 2009), probably due to the profound T-cell depletion caused by siplizumab while preserving B-cell numbers.

For the subset of PTCL patients diagnosed with systemic ALCL, brentuximab vedotin (BV) has emerged as a very interesting therapeutic approach. This antibody-drug conjugate (ADC) combines a monoclonal antibody targeted to CD30 with a potent antimicrotubule agent (monomethyl auristatin E, MMAE). CD 30 represents an ideal target in ALCL given the strong and uniform positivity. After binding to its target molecule, BV is internalized and MMAE is cleaved from the antibody molecule, exerting its action through inhibition of microtubule formation. BV was evaluated in a multicenter phase II trial for patients with relapsed systemic ALCL, at a dose of 1.8 mg/kg IV, every 3 weeks, for up to 16 cycles (Pro et al., 2012). The ORR, as assessed by an independent review committee, was reported at 86%, with a CR rate of 57%. The median durations of overall response and CR were 12.6 and 13.2 months, respectively. Significant grade 3/4 adverse events (≥10% of patients) were neutropenia (21%), thrombocytopenia (14%), and peripheral sensory neuropathy (12%). BV was approved by the FDA for treatment of systemic ALCL in patients who received at least one prior multiagent chemotherapy regimen.

Adult T-cell leukemia/lymphoma is an aggressive peripheral T-cell malignancy caused by the HTLV-I. This disease is more prevalent in geographical areas where HTLV-I is endemic, such as the Caribbean and Japan. Limited treatment options exist, and this disease carries a very poor prognosis. Most tumor cells of patients with ATL express CC chemokine receptor 4 (CCR4), which may represent an ideal target for treatment of this disease. Mogamulizumab (KW-0761), a humanized anti-CCR4 IgG1 monoclonal antibody with a defucosylated Fc region has been tested in this disease. The defucosylation of the Fc region results in enhanced ADCC, which was demonstrated *in vitro*, prompting a phase I trial in humans, which demonstrated good tolerability at the recommended dose of 1.0 mg/kg (Yamamoto et al., 2010).

A multicenter phase II trial of mogamulizumab (Ishida et al., 2012) for patients with a diagnosis of ATL whose disease had relapsed after at least one prior chemotherapy regimen was performed. Of 26 patients evaluable for efficacy, 13 achieved an objective response (ORR 50%), with eight CRs. Median PFS and OS were 5.2 and 13.7 months, respectively. The most common AE was an infusion reaction in 89% of patients, which was manageable with symptomatic medication. Mogamulizumab has been approved in Japan for the treatment of relapsed/refractory ATL and phase II studies are underway for PTCLs in Europe and the US.

### Immunomodulatory drugs

Lenalidomide has been evaluated as monotherapy for patients with relapsed/refractory PTCL in a phase II trial (Dueck et al., 2010). Twenty-four patients were enrolled and received lenalidomide at 25 mg daily, for 21 out of 28 days. The interim analysis of this study reported ORR of 30%, without any CRs achieved and a median PFS of 96 days. Lenalidomide is an oral agent, with a broad range for dose adjustments, and should be evaluated in conjunction with standard chemotherapy or other biological agents, such as bortezomib.

### Aurora kinase inhibitors

Aurora kinases are a family of oncogenic protein kinases that have a regulatory role in the cell mitotic process. Up-regulation of Aurora A kinase (AAK) has been reported in non-cutaneous PTCL, and has been correlated with rapidly dividing histologic subtypes (Mahadevan et al., 2005; Ikezoe et al., 2009; Kanagal-Shamanna et al., 2013). Alisertib (MLN8237) is a selective inhibitor of AAK, and has shown pre-clinical activity in PTCL cell lines and patient samples (Qi et al., 2013). Recently, interim results of a phase II clinical trial of alisertib in patients with aggressive B and T-cell NHL were reported (Friedberg et al., 2011). In this trial, patients were treated with alisertib at a dose of 50 mg twice daily for 7 days on 21-day cycles, until PD or unacceptable toxicities. Of the 48 enrolled patients, 17% had a diagnosis of PTCL. ORR for the whole study population was 32%, but when divided by subtype, patients with a diagnosis of PTCL presented an ORR of 57%. Currently, a phase III study of alisertib versus investigator’s choice of treatment is underway for patients with relapsed PTCL (ClinicalTrials.gov: #NCT01482962).

### Plitidepsin

Plitidepsin is a cyclic depsipeptide which has shown activity against multiple cancer cell lines, including leukemias and lymphomas. A recent, multicenter phase II trial evaluated the use of plitidepsin as a single agent for the treatment of patients with relapsed/refractory NHLs (Ribrag et al., 2013). Patients were divided into two large cohorts, including one for non-cutaneous PTCL (*n* = 34). Plitidepsin was administered as an 1 h intravenous infusion, at 3.2 mg/m^2^, on days 1, 8, and 15, every 4 weeks. Among the 29 evaluable patients with non-cutaneous PTCL, six had a response (two CRs, four PRs, ORR 20.7%). Most adverse events were mild and transient, and easily managed by dose adjustments.

### Tyrosine kinase inhibitors

Several groups have described aberrant tyrosine kinase signaling in PTCL cell lines (Piccaluga et al., 2005, 2007; Huang et al., 2010; Piva et al., 2010). Particularly, PTCL-NOS has been found to over-express constitutively phosphorylated platelet derived growth factor receptor alpha (PDFGRA). These findings have led to trials of Tyrosine kinase inhibitors (TKIs) in PTCL, since PDGFRA is one of multiple targets of this class of drugs. Initial findings of a phase I/II trial of dasatinib in relapsed/refractory NHL were reported (William et al., 2010). This study enrolled 27 patients with NHL, with relapsed or refractory disease after at least one prior systemic therapy. For the 19 evaluable patients, the ORR was 32%, with a median 2-year PFS and OS of 13 and 50% respectively. The only two patients who presented a CR had a diagnosis of PTCL, and remained alive and disease free for at least 2 years after treatment. An additional patient with PTCL achieved a partial response.

## Discussion

Peripheral T-cell lymphomas comprise a heterogeneous group of NHLs, with different clinical and biological behaviors, but with a common denominator of poor overall prognosis and lack of potentially curable approaches outside of the realm of stem-cell transplantation. Excluding ALK+ anaplastic large cell lymphoma, which is associated with a good prognosis (5-year OS 70%), and ALK-negative anaplastic large cell lymphoma (5-year OS 49%), the overall 5-year survival for other common subtypes of peripheral T-cell lymphomas is about 30–35%. However, in the last few years there have been an increasing number of trials in PTCL, and a number of novel agents are now approved in the relapsed/refractory setting, which hopefully will change the survival rates over the next few years, once these agents are more widely used by the medical community. Also, new insights into the molecular pathogenesis of PTCL, may lead to studies focusing on newer targeted agents.

Future endeavors for the treatment of PTCL should focus on developing trials specific for different subtypes, and exploring novel combination therapies in the frontline setting.

## Conflict of Interest Statement

Barbara Pro has received honoraria and a research grant from Seattle Genetics. Barbara Pro has received a research grant from Allos Therapeutics. The authors have no other relevant affiliations or financial involvement with any organization or entity with a financial interest in or financial conflict with the subject matter or materials discussed in the manuscript apart from those disclosed. No writing assistance was utilized in the production of this manuscript.
